# Feeding, Eating, and Emotional Disturbances in Children with Avoidant/Restrictive Food Intake Disorder (ARFID)

**DOI:** 10.3390/nu12113385

**Published:** 2020-11-04

**Authors:** Sharon Iron-Segev, Danielle Best, Shani Arad-Rubinstein, Martin Efron, Yaffa Serur, Hadar Dickstein, Daniel Stein

**Affiliations:** 1 Faculty of Agriculture, Food and Environment, Institute of Biochemistry, Food Science and Nutrition, School of Nutritional Sciences, Hebrew University of Jerusalem, Rehovot 7600001, Israel; dzgeller4@gmail.com; 2 Peres Academic Center, School of Nutritional Sciences, Rehovot 7610202, Israel; 3 Pediatric Psychosomatic Department, Edmond and Lily Safra Children’s Hospital, Chaim Sheba Medical Center, Tel Hashomer 5262000, Israel; shaniru5@gmail.com (S.A.-R.); Martin.Efron@sheba.health.gov.il (M.E.); yaffa.serur@sheba.health.gov.il (Y.S.); hadardi@gmail.com (H.D.); prof.daniel.stein@gmail.com (D.S.); 4 Sackler Faculty of Medicine Tel Aviv University, Tel Aviv 6997801, Israel

**Keywords:** avoidant/restrictive food intake disorder, ARFID, children, screening tool, eating, feeding

## Abstract

Avoidant/restrictive food intake disorder (ARFID) is a relatively new diagnostic category. We sought to determine whether the Stanford Feeding Questionnaire (SFQ), an instrument for assessing picky eating, can differentiate children with ARFID from control children, and whether children with ARFID would show more nonfeeding/eating emotional problems than controls. Fifty children with ARFID were compared to 98 controls. Parents completed the SFQ, Screen for Child Anxiety Related Emotional Disorders (SCARED), Strength and Difficulties Questionnaire (SDQ), and Sensory Responsiveness Questionnaire (SRQ). On the SFQ, 12 items represented child ARFID behaviors (SFQ-ARFID Scale), and another 15 items represented parental feeding problems (SFQ-PFP Scale). We found that the SFQ-ARFID and SFQ-PFP Scale scores were significantly higher in children with ARFID vs. controls. Children with ARFID demonstrated higher SDQ-Total-Difficulties, higher SDQ-Internalizing-Difficulties and lower SRQ-Hedonic scores compared with controls. Of all parameters, the SFQ-ARFID Scale best differentiated children with ARFID from control children (area under receiver operating characteristics curve = 0.939, 95% CI, 0.895–0.983, *p* < 0.001). These findings suggest that parental reports show more eating problems and emotional disturbances in children with ARFID vs. controls, and more parental feeding problems. Further research is required to determine whether the SFQ-ARFID Scale may serve as an effective screening tool for the identification of ARFID.

## 1. Introduction

Avoidant/restrictive food intake disorder (ARFID) is a relatively new diagnosis in the Diagnostic and Statistical Manual of Mental Disorders (DSM-5) [1] and the International Classification of Diseases 11th Revision (ICD-11) [2]. It represents a reformulation and expansion of the provisional diagnosis of Feeding Disorder of Infancy and Early Childhood, presented in the DSM-IV [3]. ARFID captures a varied presentation of feeding and eating disturbances leading to nutritional deficiencies, failure to meet nutritional and/or energy needs, and related psychosocial functioning imbalances [1]. In this new version of the diagnosis, symptoms of avoidance and restrictive eating are recognized as unrelated to willful weight loss, dieting behaviors, and body image disturbances. Although ARFID commonly makes a first appearance during childhood, it may occur across the entire lifespan [4].

At present, limited data is available about the incidence and prevalence of ARFID in the general population [5,6]. This is because, as a relatively new defined disorder, it has not yet been assessed sufficiently in large-scale population-based epidemiological studies [4]. Nonetheless, a school-based study assessing 8- to 13-year-old children found that 3.2% of the participants reported features of ARFID [7]. Another large-scale population-based survey in Australian adolescents and young adults found a three-month prevalence of ARFID of 0.3% [8]. Not surprisingly, the prevalence of ARFID in psychiatric clinical health-care settings such as eating disorder (ED) clinics is much higher, ranging from 5% to 22% [9,10,11,12,13]. In contrast, a study conducted in pediatric gastrointestinal clinics in the Boston area reported a much lower ARFID prevalence of only 1.5% [14].

Avoidant/restrictive eating is associated with a wide range of health problems, including malnutrition [11]; amenorrhea in older girls [15]; electrolyte abnormalities such as hypokalemia, bradycardia, and prolonged QT interval [16]; vitamin and mineral deficiencies [17]; poor linear growth [18]; dependence on tube feeding or high-energy food supplements to meet energy needs; and hospitalization for nutritional rehabilitation [19]. In addition, ARFID often co-occurs alongside other psychiatric disturbances, including anxiety disorders, particularly generalized anxiety disorder [10,11], depressive disorders, attention deficit hyperactivity disorder (ADHD) [13], autism spectrum disorder [13,20,21], and behavioral measures of disturbed sensory modulation sensitivities [22]. At present, there is insufficient data regarding the long-term course and outcome of ARFID because of the paucity of longitudinal follow-up studies [4].

Research on psychopathology of ARFID is limited by the lack of tools to identify these eating behaviors [23]. Moreover, systematic identification of ARFID, as a relatively new diagnostic entity, is critical for ensuring adequate communication among clinicians and researchers [24]. For this purpose, several tools have been developed to identify ARFID. They include the nine-item Avoidant/Restrictive Food Intake Screen (NIAS) [23], assessing avoidance of eating related to either the sensory properties of food, poor appetite/interest, or fear of negative food-related consequences, that was tested in adults; the Eating Disorders in Youth-Questionnaire (EDY-Q) [7], a 14-item instrument for assessing early-onset restrictive eating disturbances in 8–13 year old children via self-report, based on the DSM-5 criteria of ARFID; the Eating Disorder Assessment for DSM-5 (EDA-5) [25] a semi-structured interview meant to assist in the assessment of a feeding or eating disorder or related conditions according to DSM-5 criteria, and the Pica, ARFID, and Rumination Disorder Interview (PARDI) [24], a multi-informant, semi-structured interview of feeding disorders across the lifespan.

We employed a different design. ARFID, particularly in younger patients, may present both as a parent-related feeding disorder and as a child-related eating disorder. This is of importance, because treatment strategies for these two types of disorders may differ [26]. The Stanford Feeding Questionnaire (SFQ) [27,28], is a 62-item parent-reported evaluation, originally designed to assess picky eating, but also addressing many of the characteristics of ARFID. Specifically, it includes 12 items directly assessing the severity of child-related eating disturbances, and another 15 items directly assessing the severity of parental feeding problems. Hence, we opted to find out whether the use of these two SFQ subscales would differentiate between children diagnosed with ARFID and control children.

Specifically, we aimed to evaluate whether the use of the SFQ subscales assessing children’s problematic eating and parents’ problematic feeding, would have the potential to support a clinical diagnosis of ARFID obtained according to two sets of criteria: (a) the DSM-5 criteria for ARFID [1] and (b) a nutritional assessment based on Bryant-Waugh’s diagnostic checklist for ARFID [29]. This assessment checks the child’s eating and feeding and is based on the DSM-5 Criterion A of ARFID [1], i.e., avoidance of food, not related to dieting/body image disturbances, interfering with the child’s medical condition and psychosocial functioning. In addition, we sought to determine whether children with ARFID would differ from control children in their overall psychiatric condition and in several specific domains (anxiety, sensory modulation) previously shown to be more disturbed in children with ARFID [15,27]. Thus, the following were the hypotheses of the present study:

We hypothesized that the eating- and feeding-related subscales of the SFQ, would distinguish children diagnosed with ARFID from control children based on the two previously mentioned methods. Additionally, regarding nonfeeding/eating emotional problems, we hypothesized that the ARFID group would show less favorable overall psychiatric conditions, greater anxiety, and more disturbed sensory responsiveness compared with the control group.

## 2. Materials and Methods

### 2.1. Participants and Procedures

A cross-sectional study was conducted over 2.5 years (1 January 2017 to 30 June 2019) in the outpatient pediatric eating disorders (ED) clinic, located in the Edmond and Lily Safra Children’s Hospital, the Chaim Sheba Medical Center, Tel Hashomer, Israel. Sample size was calculated using a significance level of 5%, a power of 80%, and a 1:2 ratio of ARFID to control groups. Thus, to identify a medium effect size (Cohen’s d = 0.5), our sample required 48 children who were diagnosed with ARFID and 96 children with typical development [30].

The ARFID group comprised of 50 families of children referred to our clinic during the study period who received an ARFID diagnosis from our psychiatric team and whose parents agreed to participate in the study. These 50 children (Mean age = 9.53, SD = 2.41; 55% males) represented 20% of all 240 patients referred to our clinic during the targeted 2.5-year period. The remaining 80% were either children with ARFID not included in the study (*n* = 30), or youngsters diagnosed with anorexia nervosa, bulimia nervosa, or binge-eating disorder (*n* = 160). The children with ARFID that did not participate in this study (*n* = 30), did not differ in age, sex, parents’ education level, and family’s socioeconomic status (results not shown). The children with ARFID were excluded from the study due to parental refusal to take part (*n* = 20), or because they did not meet the study’s inclusion criteria (a satisfactory knowledge of the Hebrew language, or children with medical disturbances that could affect their eating and feeding; *n* = 10).

For the ARFID group, children’s height and weight measurements were taken by experienced clinical dietitians in the morning hours, following a night’s fast and passing of urine, according to standardized procedures [31]. Body mass index (BMI) was based on the formula weight (kg)/height (m)^2^. BMI standard deviation scores (z-scores) were calculated using age and sex-specific growth data (based on the Centers for Disease Control and Prevention’s Year 2000 Growth Charts), confirmed for assessing Israeli children and adolescents [32]. Regarding additional psychiatric disorders in the ARFID group, 18 children (36%) had evidence of other psychiatric disorders: depressive disorders (*n* = 2), anxiety disorders (*n* = 5), obsessive compulsive disorder (*n* = 2), post-traumatic stress disorder (*n* = 1), and ADHD (*n* = 10). Two of these 18 children had two additional psychiatric disorders.

The control group (*n* = 98), comprised of families of typically developing children (Mean age = 7.74, SD = 2.32; 45% males), was a convenience sample recruited from several different communities in Israel that were similar in their regional distribution to the research group. Using a snowball sampling method, small groups of mothers were invited to hear a presentation by experienced registered dietitians (S.I-S. and D.B.) on current dietary recommendations for children. These lectures were conducted in a community setting. At the end of the lectures, mothers were asked to complete the parent-report questionnaires as well as a structured demographic and health questionnaire. Control families were excluded from the study if the children had been previously diagnosed with eating-related disturbances, psychiatric disturbances, or physical or neurological illnesses (e.g., diabetes mellitus, thyroid disorders) that could potentially affect food consumption and weight. We did not assess the control children for height and weight.

The study was approved by the Sheba Medical Center Human Subject Committee (42759; May 2014) for the research participants, and the Ethics Committee for Research in Human Subjects, the Robert H. Smith Faculty of Agriculture, Food and Environment, the Hebrew University of Jerusalem, Rehovot, Israel, (AGHS; May 2018) for the control participants. All parents agreed to participate in the study by signing a written informed consent after receiving explanation about the study’s aims, methods, and requirements.

### 2.2. Assessment of Participants

#### 2.2.1. Psychiatric Assessment

Parents of children with ARFID were independently interviewed by two experienced child and adolescent psychiatrists (D.S. and Y.S.) using a semi-structured interview, based on the DSM-5 [1] ARFID criteria. Only children for whom a diagnosis of ARFID was achieved with this interview were included in the current study. It is of note that the diagnosis of ARFID according to the DSM-5 does not differentiate among subtypes of avoidance of eating related to either the sensory properties of food, poor appetite/interest, or fear of negative food-related consequences. Our clinic requires that all children be in elementary school above the age of 6 years; therefore, most had longstanding eating problems (except for children developing problematic eating following pharyngeal-related traumas). Moreover, the psychiatrists mostly received previous relevant data, provided by pediatricians, clinical dietitians, and/or other treatment providers.

Other psychiatric diagnoses in children with ARFID were assessed by the two psychiatrists with interviewing the parents using the Structured Clinical Interview for DSM-IV Axis I Disorders (SCID)—Patient Edition [33], adapted for DSM-5 [1]. Diagnosis of comorbid ADHD was obtained using the ADHD module of the Schedule for Affective Disorders and Schizophrenia for school-age children—present and lifetime version (K-SADS-PL) [34]. In this study, we used the SCID-I/P Version 2.0 [33] rather than the K-SADS-PL [34] to diagnose other psychiatric disorders, because although we were investigating children, the parents were our interviewees (there is no ADHD module in the SCID-I/P Version 2.0). All ARFID and other psychiatric diagnoses achieved by the two psychiatrists were confirmed in clinical meetings of the multi-professional team of the clinic. Control parents were contacted by telephone and interviewed by the registered dietitians (S.I-S., D.B.) to determine if the child met inclusion criteria, as described above. If these criteria were met, the researcher scheduled a meeting with the parent to attend a lecture and to fill out the study’s questionnaires.

#### 2.2.2. Nutritional Assessment

In the present study, following the psychiatric evaluation, all the parents of children with ARFID symptoms were independently interviewed by an experienced registered dietitian (S.A-R.). The nutritional assessment was based on the caloric intake and the variety and quantity of food intake. The interviewer utilized a dietary recall methodology, where parents were asked to describe the amounts and types of all food and beverages that their child had consumed in the 24 h before the interview. From this report, the dietitian estimated the child’s total daily caloric intake and the number of different types of foods consumed daily (diet variety). The list of food types in the present study was based on Bryant-Waugh’s checklist [29]. This author presented in 2013 a case study of a child with ARFID and developed a checklist of diagnostic criteria comprising of seven questions to guide practitioners in establishing whether the symptoms correspond with the definition of ARFID using DSM-5 Criterion A [1]. The seven questions focus on the variety and quantity of food intake, the persistence of the eating disturbance, and the presence of signs of nutrient deficiencies and/or of disruptions in daily life [29].

In the current study, children were considered to have ARFID if their caloric intake failed to meet the Recommended Dietary Allowances (RDA) for sex and age and/or if they consumed fewer than 15 different food types. We used Fraker et al.’s (2007) description of highly selective eating [35], referring to children who limit their diet to less than 10–15 food types. However, as children with ARFID tend not to restrict simple carbohydrates [36], we applied Fraker et al.’s definition to food types other than refined carbohydrates and ultra-processed food such as sweets. Thus, using the Bryant-Waugh checklist, we considered children to have ARFID if their total daily caloric intake failed to meet the RDA for sex and age and/or if they consumed fewer than 15 different food types (excluding from the diet variety calculation: simple refined carbohydrates and ultra-processed foods such as sweets). Comparison of the psychiatric and dietetic assessments of ARFID, we found that all fifty children diagnosed as ARFID by the registered dietitian were also diagnosed as ARFID by the psychiatrists. All ARFID diagnoses were finalized in the team meetings of the ED clinic.

### 2.3. Parent-Report Measures

#### 2.3.1. Stanford Feeding Questionnaire (SFQ) 

The SFQ [27,28] has been used to assess children’s eating behaviors and parental feeding practices, and has been previously shown to differentiate children with picky eating from healthy controls [27,37]. The study team reviewed the original version and the translation to Hebrew done for this study by professional translators. The SFQ includes 62 items asking parents to rate varied eating- and feeding-related behaviors of the child and family, such as: “Is your child a picky eater?”, “Does your child eat a very limited variety of food types?”, ”When your child is eating with the family, how frequently does he/she try to leave the table early?” or “When the family eats dinner together how often do you plan separate food for your child?”. It is of note that the SFQ does not include items assessing dietary-behaviors or body-related concerns (the absence of these problems in the ARFID group was verified in the psychiatric interview), nor does it include items differentiating among the types of ARFID.

For this study, our team proposed two subscales comprising of all SFQ items assessing the severity of (a) children’s eating behaviors (the SFQ-ARFID Scale) and (b) parental feeding problems (the SFQ-PFP Scale) according to a 7-point Likert scale ranging from 0 (nothing/never) to 6 (very much/always/all the time). This allowed for the development of numerical scales. All other items of the SFQ are verbal descriptions of different eating-related and feeding-related behaviors.

The SFQ-ARFID Scale: To evaluate the severity of ARFID eating behaviors in children with ARFID, we selected 12 items from the SFQ: #15,16,17,22,31,32,33,34,35,36,37, and 40A (see Table 1 for description). Higher mean scores for these 12 items comprising the SFQ-ARFID Scale indicated that parents of children with ARFID saw their child’s eating behaviors as more problematic than parents of control children. The internal consistency of the 12 SFQ -ARFID items was excellent (Cronbach’s alpha = 0.944).

The SFQ-PFP Scale: To evaluate the severity of parental feeding problems, we selected 15 items from the SFQ: #24, 26, 38, 39, 44, 45, 46, 47, 48, 49, 50, 59, 60, 61, and 62 (see Table 2 description). Higher mean scores for these 15 items comprising the SFQ-PFP subscale indicated that parents of children with ARFID saw their own feeding behaviors as more problematic than parents of control children. The internal consistency for the 15 SFQ-PFP items was good (Cronbach’s alpha = 0.743). The items in the SFQ-ARFID and SFQ-PFP scales were chosen by the research team based on the type and content of the item. All items assessing maladaptive eating behaviors were included in the SFQ-ARFID scale. If an item described a problematic parental feeding behavior, it was included in the SFQ-PFP scale.

#### 2.3.2. Strengths and Difficulties Questionnaire (SDQ) 

The SDQ [38] is a 25-item questionnaire assesses emotional problems, conduct problems, hyperactivity, peer problems, and prosocial behavior. Parents have rated the extent to which their child was characterized by SDQ items such as “often has temper tantrums,” “is often restless, overactive,” and “is easily distracted” on a 3-point scale: 0 (never), 1 (sometimes), or 2 (always). The SDQ-Total-Difficulties score has been calculated by summing the scores of all subscales, except for prosocial behavior. The SDQ-Internalizing Difficulties score has been calculated by summing the emotional and peer problems scores, and the SDQ-Externalizing Difficulties score has been calculated by summing the conduct problems and hyperactivity scores. The questionnaire has been previously validated in a Hebrew-speaking population [39]. The SDQ scores were analyzed as continuous variables, with higher scores indicating more difficulties. The Hebrew translation of the SDQ has been previously used in Israeli population [39]. The internal consistency of the SDQ-Total Difficulties Score (Cronbach’s alpha = 0.76), SDQ-Externalizing Score (Cronbach’s alpha = 0.70) and SDQ-Internalizing Score (Cronbach’s alpha = 0.73) for the present study was sufficient.

#### 2.3.3. Screen for Child Anxiety Related Disorders Questionnaire (SCARED) 

The SCARED Questionnaire [40,41] is used for children ages 8–18 years to screen for anxiety-related symptoms. The current study used the 66-item revised SCARED version to assess anxiety-related symptoms compatible with the differentiation of anxiety disorders according to DSM-IV [42] into panic disorder, separation anxiety disorder, generalized anxiety disorder, social phobia, specific phobia (animal phobia, situational and environmental phobia, and blood-injection-injury phobia), obsessive compulsive disorder, and traumatic stress disorder. Parents have rated the extent to which their child is characterized by SCARED items such as “When my child feels frightened, it is hard for him/her to breathe” or “My child worries about other people liking him/her”. Each question has three response choices: almost never (score = 0), sometimes (score = 1) and often (score = 2). A Total SCARED score was calculated by summing up all items, analyzed as continuous variables, with higher scores indicating greater overall anxiety [43]. The Hebrew translation of the SCARED has been previously used in Israeli populations [44]. The internal consistency of the Total SCARED Anxiety Score in the present study was excellent (Cronbach’s alpha = 0.94).

#### 2.3.4. Sensory Responsiveness Questionnaire (SRQ) 

In the SRQ [45] parents receive a set of 58 typical scenarios encountered in daily life. Each scenario involves one sensory stimulus in one modality, including auditory, visual, gustatory, olfactory, vestibular, and somatosensory stimuli (excluding pain). Items are presented in a manner that attributes to a situation either a hedonic/pleasurable valence (e.g., “My child enjoys activities where he/she is spinning, such as riding on a carouse”) or an aversive valence (e.g., “It bothers my child to get a haircut”). Parents are asked to rank the intensity and frequency at which these daily sensory experiences induce pleasure (associated with sensory-seeking behaviors resulting from sensory under-responsiveness) or aversion (associated with sensory-avoiding behaviors resulting from sensory over-responsiveness) in their child. Each item is scored twice, for intensity and for frequency. Items are scored on a 5-point Likert scale (1 = not at all, 5 = very much, and 0 = not relevant or never tried). If an item is unanswered or answered as “0”, it is not included in the calculation [46]. Higher scores indicate more intense/frequent pleasure/aversion. The interaction between the two scales (intensity × frequency) represents the combined SRQ score. The SRQ has demonstrated good psychometric quality in previous studies of Israeli samples in differentiating individuals with and without disturbed sensory modulation [46]. Our team has previously shown that the SRQ may differentiate between girls with anorexia nervosa and girls with typical development [47]. The internal consistency of the SRQ-Hedonic Score (Cronbach’s alpha = 0.72); and SRQ-Aversive Score (Cronbach’s alpha = 0.77) in the present study was good.

### 2.4. Statistical Analysis

Categorical variables were described as frequency and percentage. Continuous variables were evaluated for normal distribution using histogram and Q-Q plot. Child sex was compared between groups using chi-square testing. Continuous and ordinal variables were compared between the two groups using independent samples *t*-tests or the Mann-Whitney test. The Mann-Whitney test was used for between-group comparisons in variables regarding ARFID food variety and caloric intake per day, and in the SFQ-ARFID and SFQ-PFP Scale scores. The internal consistency of the ARFID and PFP Scales and other psychometric tools was evaluated using Cronbach alphas. Youde’s Index was used to explore for the best cutoff point of the SFQ-ARFID scale. The maximal value of the Index was set to reveal the best cutoff point. In addition, Sensitivity, Specificity, Negative Predictive Value, Positive Predictive Value, Accuracy, Positive Likelihood Ratio (LR^+^), and Negative Likelihood Ratio (LR^-^) were calculated. The ability of the questionnaires to distinguish between children with and without ARFID was evaluated using the area under the receiver operating characteristics (AUROC) curve. Spearman’s correlation coefficients were used to study correlations between the BMI *Z*-score and the SFQ-ARFID and SFQ-PFP Scale scores and between the parent psychometric reports and the children’s age, and for the correlations among the SFQ-ARFID Scale and the other psychometric scales. When the child’s age was found to correlate significantly with a parent report, we used an analysis of covariance (ANCOVA) to control for age when comparing the two studied groups. All statistical tests were two-sided and *p* < 0.05 was considered statistically significant. SPSS software (IBM SPSS Statistics for Windows, version 23, IBM cooperation, Armnok, NY, USA) was used for all statistical analyses.

## 3. Results

A total of 148 children participated in the study. Fifty children were diagnosed with ARFID and 98 were community controls. Mean age of the children with ARFID and control children was 9.53 (SD = 2.41) and 7.74 (SD = 2.32) respectively (*p* < 0.001). There was no difference in sex ratio between the groups, with 55% males in the ARFID group and 45% in the controls (*p* = 0.907).

### 3.1. Between-Group Differences in Eating and Feeding Related Parameters

The findings of this study showed that the score of the proposed SFQ-ARFID Scale, based on 12 selected items from the SFQ describing maladaptive eating-related behaviors, was significantly higher for children with ARFID as diagnosed in the psychiatric evaluation [Mean (SD) 4.46 (1.01) than for control children [Mean (SD) 1.89 (1.18)], (*p* < 0.001). In addition, the score of the proposed SFQ-PFP Scale describing 15 maladaptive parental feeding practices, was significantly higher for ARFID children [Mean (SD) 1.63 (0.56)] compared to controls [Mean (SD) 1.42 (0.68)], (*p* = 0.013). A weak correlation was found between the child’s age and the SFQ-ARFID Scale score (r = 0.303, *p* < 0.001). After controlling for the child’s age, the between-group difference in SFQ-ARFID Scale score (*p* < 0.001) was still significant.

The maximal value of the Index was set to reveal the best cutoff point for total SFQ-ARFID Scale and showed a cutoff point of 3.46 with a sensitivity of 88% (95% CI: 76–95%), and a specificity of 90% (95% CI: 83–96%); Positive Predictive value was 83% (95% CI: 70–92%); Negative Predictive Value was 94% (95% CI: 87–98%); Accuracy was 90% (95% CI: 84–94%); Positive Likelihood Ratio (LR^+^) was 9.58 (95% CI: 5.10–18.00); and Negative Likelihood Ratio was (LR^-^) 0.13 (95% CI: 0.06–0.28).

A detailed analysis of the separate SFQ-ARFID Scale items revealed that all twelve items independently differentiated between children with ARFID and control children (see Table 1). In contrast, when examining all 15 items of the SFQ-PFP Scale, only three items showed higher problematic parental feeding behaviors in parents of children with ARFID vs. control children (see Table 2).

### 3.2. Additional Eating and Feeding-Related Findings in Patients with ARFID

No significant correlations were found between the Total SFQ-ARFID Scale score and the dietitian’s assessment of both caloric intake/day (*p* = 0.945) and food variety/day (*p* = 0.600). Similarly, there was no significant relationship between the Total SFQ-PFP Scale score and the dietitian’s assessment of both caloric intake/day (*p* = 0.293) and food variety/day (*p* = 0.173). Regarding the influence of other psychiatric disorders, we found no significant differences in SFQ-ARFID Scale score when comparing ARFID children with additional psychiatric disorders (*n* = 18) to children with only ARFID (*n* = 32; *p* = 0.716). Last, no significant correlations were found in the research group between the BMI Z-score and the SFQ-ARFID Scale (*p* = 0.793), and SFQ-PFP Scale scores (*p* = 0.404).

### 3.3. Between-Group Differences in Non-ED-Related Parameters

The comparison between the research and control groups in the non-ED related psychometric tools is summarized in Table 3. Children with ARFID scored higher than control children on SDQ-Total Difficulties score and SDQ-Internalizing Difficulties score, and lower in the SRQ-Hedonic scale, i.e., reduced sensory-seeking behaviors related to sensory under-responsiveness. No between group differences were found for the SDQ-Externalizing Difficulties score, Total SCARED anxiety score, and SRQ-Aversive score. A weak correlation was found between the child’s age and the SDQ Internalizing Difficulties score (r = 0.169, *p* = 0.048). After controlling for the child’s age, the between-group difference in SDQ-Internalizing Difficulties score (*p* < 0.001) was still significant.

### 3.4. The Ability of the Different Psychometric Tools to Differentiate Children with ARFID from Control Children

**Figure 1:**
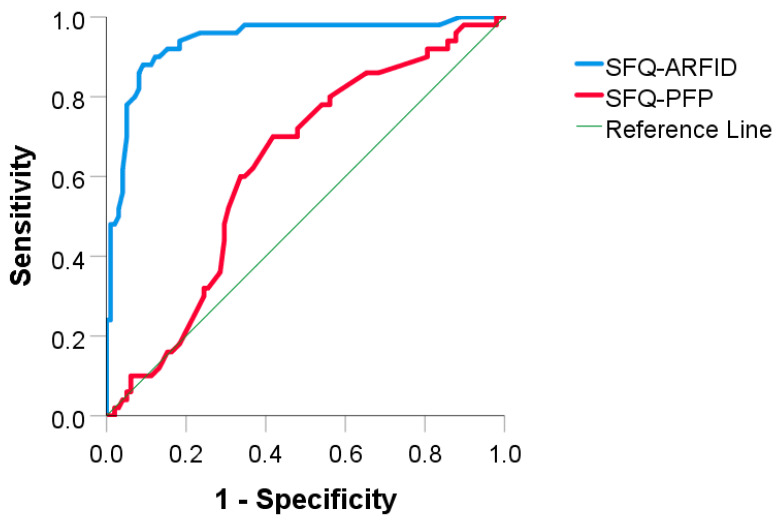
The ability of the scales of SFQ-ARFID and SFQ-PFP to distinguish between the ARFID and control groups using the area under receiver operating characteristics curve (AUROC). Note. ARFID = avoidant/restrictive food intake disorder; SFQ = Stanford Feeding Questionnaire; PFP = Parental Feeding Problems; AUROC = area under receiver operating characteristics curve.

Table 4 and Figure 1 describe the potential of the parent’s reports to differentiate children in the ARFID group from children in the control group, using the area under the ROC curve (AUROC) analysis. An area of 1 represents a perfect result, and an area of 0.5 represents a nonsignificant result. Using this AUROC analysis, the SFQ-ARFID Scale was “excellent” in supporting a psychiatric interview-based diagnosis of ARFID. The AUROC is equivalent to the probability that a randomly chosen ARFID child will be ranked higher in the scale than a randomly chosen control child. The SFQ-PFP Scale showed only a “fair” accuracy for supporting the clinical diagnosis of ARFID [48].

Table 5 summarizes the correlations found between the different psychometric scales and the SFQ-ARFID Scale. Significant correlations were found for the SDQ-Total Difficulties Scale and the SDQ-Internalizing Scale for all children, but not for the ARFID and control children separately, and for the SFQ-PFP Scale, for all children, as well as for the ARFID and control children separately.

## 4. Discussion

The primary aim of the present study was to determine if the SFQ could distinguish between children diagnosed with ARFID using the DSM-5 criteria [1] from control children. Specifically, we examined whether two proposed subscales of the Stanford Feeding Questionnaire [27,28], evaluating the child’s maladaptive eating behaviors (SFQ-ARFID Scale) and the parents’ maladaptive feeding practices (SFQ-PFP Scale), would support the differentiation between ARFID and control children according to our center’s standard psychiatric interview procedure based on the DSM-5 criteria [1] and according to a dietetic assessment using Bryant-Waugh’s diagnostic checklist [29]. In addition, we sought to determine whether children diagnosed with ARFID in our study would show elevated scores on measures of emotional and behavioral disturbances previously shown to be more disturbed in children with in ARFID.

### 4.1. Eating- and Feeding-Related Findings

In line with our first hypothesis, children diagnosed with ARFID using *DSM-5* criteria [1] were found to score higher on both the SFQ-ARFID Scale and the SFQ-PFP Scale, compared to typically developing children. These findings suggest that, according to the parents, the clinically referred children exhibited not only the expected higher rates of eating problems but also the parents themselves reported enacting more maladaptive feeding patterns compared to parents in the control group. These findings support a critical review published by Kennedy at al. [26], suggesting that ARFID is characterized by features applicable to both maladaptive eating and maladaptive parental feeding patterns [26].

The significant correlation found between the SFQ-ARFID Scale and the SFQ-PFP Scale lends further support for the likelihood of an association between the parents feeding patterns and the child’s eating behaviors, both for normally-developing children and for children with ARFID. Nonetheless, because our study was cross-sectional, it is not possible to determine whether in these families the child’s problematic eating behaviors may result from problematic parental feeding patterns, or whether long-standing eating disturbances in children may increase parents’ stress and anxiety, eventually culminating in problematic feeding patterns. A future large-scale prospective longitudinal study might provide adequate answers for these issues.

The proposed SFQ-ARFID scale assessing parental report of problematic eating behaviors in their children was found to be a short and easily completed tool that can serve as a screening tool for the identification of children with ARFID. Thus, all 12 items of this scale have distinguished between children who were clinically diagnosed with ARFID using the DSM-5 [1] and a nonclinical group of children. The high sensitivity and specificity of the cutoff point of the SFQ-ARFID Scale lends further support for its potential as a screening tool. Nevertheless, the SFQ-ARFID scale has two important drawbacks that should be addressed. It does not include items related to dieting behaviors and body image aspects, nor to the different types of food avoidance that can be included under the ARFID category. Additional studies should be implemented to find out whether the SFQ-ARFID Scale can serve as a first step in screening parents of children with potential ARFID, suggesting which parents require further evaluation.

The findings are less robust for the 15-item SFQ-PFP Scale, assessing the parents’ perceptions of their own feeding-related behaviors. Although the SFQ-PFP Scale differentiates between the research and control groups, only three of its 15 items have shown significant between-group differences, and AUROC analysis yielded only a fair result. The strength of the SFQ-ARFID Scale relative to the SFQ-PFP Scale suggests that parents may possibly be more accurate informants of their children’s problematic eating behaviors than of their own problematic feeding patterns. Alternatively, from a methodological point of view, all 12 SFQ-ARFID Scale items seem to precisely capture the specific avoidant/restrictive clinical presentation of the disorder. By contrast, many items included in the SFQ-PFP Scale seem to represent relatively nonspecific feeding behaviors (e.g., offering food as a reward or for soothing, see Table 2). These behaviors may be used by frustrated parents in dealing with any type of disturbed eating, as well as in more general conflictual relationships with their children.

From a different angle, the SFQ-ARFID and SFQ-PFP Scales have not been correlated in the ARFID group with the children’s BMI-SDS scores. This finding may be associated with the lack of inclusion of reduction in weight as a necessary criterion for the diagnosis of ARFID [1]. Furthermore, it should be readdressed in future studies in larger populations.

As noted earlier, several tools have been previously proposed for the identification of ARFID. In contrast to the EDA-5 [25], and the PARDI [24], the SFQ-ARFID Scale is a short parent-reported scale, rather than a semi-structured interview, and in contrast to the EDY-Q [7], it is completed by the parents rather than by the children. It may provide a new tool based on its potential to serve as a quick and easy screening tool.

No significant correlations were found between the SFQ-ARFID Scale and the SFQ-PFP Scale scores and the assessment by the clinical dietitian of both caloric intake/day and food variety/day. These results were unexpected, because all children identified with ARFID according to the DSM-5 [1] psychiatric assessment were also identified as such in the dietetic assessment. They were further unexpected because usually, nutritional assessments indicate that children with ARFID eat fewer calories per day and their variety of food intake is more limited that of control children. Thus, Harshman et al., (2019) [36] compared the eating patterns of children, adolescents and young adults (age 9–22 years) with full or sub-threshold ARFID to controls, using a four-day food record. They found that participants with ARFID consumed higher refined carbohydrate processed foods, total carbohydrates, and added sugars, and lower protein, vegetables, and vitamins K and B12. If the parents in our study were required to report four-day food records, completed at home prior to the first meeting with the clinical dietitian (including food records of week-days and weekends) the amount of daily intake of calories and variety of food could have been estimated more precisely than using a single 24 dietary recall performed during the dietetic assessment [49]. In addition, our decision to define the cutoff point for low vs. normal consumption of a variety of foods each day as 15 different food items (excluding refined carbohydrates and ultra-processed foods such as sweets) [35,36], should be reconsidered, being perhaps overly strict. In this respect, it is of note that the clinical diagnosis of ARFID by the psychiatrists was based on a general impression of the parents over a longer period, rather than on a detailed list of the food consumed, as well as on previous relevant material.

### 4.2. Overall Non-ED Related Emotional Problems

The second hypothesis of our study was partially confirmed. Thus, according to their parents, children diagnosed with ARFID exhibited significantly more pathological results than the control group in their overall difficulties (SDQ-Total) as well as in their internalizing problems, but not in their externalizing problems. The SDQ-Internalizing Difficulties finding, suggesting that children with ARFID may have more emotional and social problems, is not surprising, because psychosocial difficulties associated with eating and feeding difficulties are included among the DSM-5 [1] diagnostic criteria for ARFID. The correlations found between the SFQ-ARFID Scale and both the SDQ-Total Difficulties and SDQ-Internalizing Difficulties scales lend further support for this contention. Moreover, previous studies have noted the association between ARFID and internalizing disturbances [50,51], showing that the avoidance and reduced nutritional intake of children with ARFID might highly interfere with their overall psychosocial functioning [52,53].

However, as mentioned above, our findings also show that in contrast to the results on the SDQ-Internalizing Difficulties scale, children with ARFID did not show greater anxiety as rated by parents on the SCARED scale. Moreover, the AUROC analysis pinpointed the SDQ-Internalizing Difficulties scale as showing “good” accuracy for differentiating children with ARFID from control children, whereas the SCARED scale did not distinguish between the two groups. One reason for this discrepancy might be methodological. The five-item SDQ-Internalizing scale encompasses physical complaints, worries, unhappiness, nervousness in new situations, and many fears. By contrast, the SCARED assesses only anxiety related symptoms. Nonetheless, other studies on children with ARFID have emphasized not only their anxious/avoidant characteristics [52] but also their high rate of anxiety disorders [53], and anxiety is a prominent feature in children with ARFID [1]. As noted earlier, the percentage of anxiety disorders in our ARFID sample did not differ from findings in community cohorts. The apparent difference in the anxiety profile of our children from other young ARFID populations awaits further investigation.

The lack of significant between-group differences for the SDQ-Externalizing Difficulties scale was unexpected, as ARFID was previously associated with elevated rates of ADHD [13,54], although ADHD is not synonymous with externalizing difficulties. Five of the 10 item of SDQ-Externalizing Difficulties scale, relate to hyperactivity or inattention. Moreover, in the present cohort, 20% (10/50) of the children with ARFID had an additional diagnosis of ADHD, a rate surpassing the prevalence in community populations [42]. This negative finding awaits further investigation

Regarding the sensory processing issues that we expected to correlate with ARFID, our findings partially supported our second hypothesis. Thus, children with ARFID did demonstrate significantly lower SRQ-Hedonic scores, associated with a reduction in sensory-seeking behaviors resulting from sensory under-responsiveness, compared to control children. However, unexpectedly, no between-group differences emerged for the SRQ-Aversive score, associated with sensory-avoiding behaviors related to sensory over-responsiveness. The latter finding is unclear, considering that disgust from food is considered a prominent feature in the development and maintenance of ARFID [55] and is likely associated with elevated food aversion related to hypersensitivity to taste, smell, and/or food texture [51,56,57]. However, in support of our SRQ-Hedonic finding, one study in an adult community sample found that picky eating was related not only to elevated food aversion but also to reduced pleasure from food [51].

### 4.3. Study Limitations

The findings of our study should be regarded as preliminary and interpreted with caution because of several limitations. First, as our study was cross-sectional, it was not possible to establish causal relationships between problematic parental feeding behaviors, as well as noneating related psychopathology in the children, and the development and maintenance of ARFID. Second, as we did not assess the nutritional intake of the control children, we could not compare the daily caloric intake and food variety between the two groups. In addition, since weight and height measures were not reported by parents of control children, we could not compare the BMI SDS score of the patients to that of the controls. Third, as our study relied on parental report, this may have affected the results of older participants, less likely to be under close supervision of their parents. Fourth, as the SFQ-ARFID and the SFQ-PFP Scales were developed in our clinic, thus, they have not been studied in non-Hebrew-speaking populations. Therefore, we have yet no knowledge whether the SFQ-ARFID Scale can serve as a potential screening tool in other populations. Lastly, the clinical interview carried out by the psychiatrists, although relying on the DSM-5 [1] criteria and on previous relevant confirmatory material, was open-ended and not standardized, and the independent dietetic assessment, although comprehensive and standardized, had several methodological flaws, such as only 24-h dietary recall assessment.

### 4.4. Advantages, and Directions for Future Research

Advantages of this hypothesis-generated study include the use of both clinical and nutritional interviews to diagnose ARFID, as well as parental reported scales to compare between children diagnosed with ARFID and children with typical development. Furthermore, a thorough investigation of the SFQ items enabled us to develop a novel brief, practical screening tool that could be potentially used to screen patients for further evaluations. The use of the cutoff point of the SFQ-ARFID Scale found in our study for this purpose could greatly assist in such decisions.

Another important finding of this study is that elementary-school children with ARFID already exhibit considerable psychiatric morbidity. The finding that over a third of these children (37.5%) had another psychiatric disorder in addition to ARFID is striking, although some studies have shown even higher rates of other psychiatric disorders in children with ARFID, such as 45% in Kambanis et al., [52], assessing both children and adolescents and 75% in Keery et al., [58], assessing children with a mean age of 12.4 years. Nonetheless, no differences were found in SFQ-ARFID Scale score between ARFID children with vs. without other psychiatric disorders. This might be, perhaps, because in such young children, the presence of additional psychiatric morbidity did not yet affect the severity of the disturbed eating.

There is a need to extend the study of the utility of the SFQ-ARFID Scale. Prospective longitudinal, studies using the SFQ-ARFID Scale in both parents and participants of different ages, will show if the tool can also be used for assessing possible changes in the severity of ARFID symptoms over time. Lastly, there is a need to examine whether the SFQ-ARFID Scale can differentiate children with ARFID from children with other types of avoidant restricting eating, e.g., picky eating, and from non-ARFID disordered eating.

## 5. Conclusions

This study suggests that SFQ-ARFID Scale as completed by parents, has the potential to differentiate children with ARFID from normally-developing children. It may serve as a screening tool in the identification of the disorder. Our findings further suggest that children with ARFID demonstrate not only disturbances in eating and parental feeding behaviors, but also decreased overall psychosocial functioning, and elevated internalizing emotional difficulties. These findings support previous studies showing that ARFID is not just an eating and feeding disorder, but is often associated with additional psychopathology.

## Figures and Tables

**Table 1:** Between-group differences of the 12 items in the proposed SFQ-ARFID Scale.

SFQ-ARFID Items	ARFID (*n* = 50) Mean (SD)	Control (*n* = 98) Mean (SD)	*p*-Value	Effect Size D
SFQ 15: Do you get upset if your child eats too little?	4.81 (1.41)	2.09 (1.89)	**<0.001**	**1.63**
SFQ 16: Do you worry that your child is currently underweight?	3.78 (2.17)	0.75 (1.29)	**<0.001**	**1.70**
SFQ 17: Do you worry that your child will become underweight?	3.94 (1.90)	0.72 (1.23)	**<0.001**	**2.01**
SFQ 22: Is it difficult to get your child to eat new foods?	5.20 (1.54)	3.08 (1.89)	**<0.001**	**1.23**
SFQ 31: Is your child a picky eater?	5.10 (1.58)	2.88 (1.96)	**<0.001**	**1.25**
SFQ 32: Do you make special meals for your child because he/she is a picky eater?	4.58 (1.80)	2.05 (2.06)	**<0.001**	**1.31**
SFQ 33: Is it a struggle to get your child to eat?	3.62 (1.88)	1.35 (1.52)	**<0.001**	**1.33**
SFQ 34: Does your child frequently have a poor appetite?	3.78 (1.66)	1.38 (1.27)	**<0.001**	**1.62**
SFQ 35: Do you get upset if your child does not eat enough?	4.78 (1.54)	1.75 (1.76)	**<0.001**	**1.83**
SFQ 36: Does your child eat a very limited variety of food?	5.02 (1.58)	2.19 (1.83)	**<0.001**	**1.66**
SFQ 37: Will your child only eat foods if they are prepared in a specific way?	4.46 (2.05)	2.22 (1.90)	**<0.001**	**1.13**
SFQ 40A: Does your child often express a strong dislike for a food?	4.44 (1.59)	2.21 (1.66)	**<0.001**	**1.37**

Note: SFQ—Stanford Feeding Questionnaire; ARFID—Avoidant restrictive food intake disorder; SD—Standard deviation. Bold number represents statistically significant results.

**Table 2:** Between-group differences of the 15 items in the proposed SFQ-Parental Feeding Problems Scale.

SFQ-PFP Items	ARFID (*n* = 50) Mean (SD)	Control (*n* = 98) Mean (SD)	*p*-Value	Effect Size
SFQ 24: Do you offer foods your child likes (for example: candy, ice cream, cakes, pastries) as a reward for eating foods that are good for him/her?	1.56 (1.64)	1.33 (1.72)	0.268	0.14
SFQ 26: Do you make your child finish all his/her dinner before she/he can have a dessert?	2.74 (1.87)	2.81 (2.14)	0.868	−0.03
SFQ 38: When the family eats dinner together do you often plan a separate food for your child?	4.20 (1.90)	1.94 (1.99)	**<0.001**	**1.16**
SFQ 39: Do you argue with your spouse about your child’s eating habits or food selection?	1.90 (1.86)	1.32 (1.45)	0.096	0.35
SFQ 44: Do you often soothe your child by giving him/her something to eat or drink	1.49 (1.56)	1.43 (1.42)	0.937	0.04
SFQ 45: Do you often give your child something to eat or drink if he/she is bored, even if you think he/she is not hungry?	1.00 (1.34)	0.89 (1.18)	0.777	0.09
SFQ 46: Do you often give your child something to eat to stop a temper tantrum?	0.74 (1.31)	0.78 (1.16)	0.587	−0.03
SFQ 47: Do you offer your child his/her favorite foods in exchange for good behavior?	0.80 (1.34)	1.94 (1.39)	0.026	−0.84
SFQ 48: Do you use food to occupy your child while you are attending to other matters?	0.20 (0.50)	0.57 (0.99)	**0.009**	−0.47
SFQ 49: At family meals do you let your child choose the foods he/she wants from what is served?	0.52 (1.13)	0.71 (1.26)	0.488	−0.16
SFQ 50: Do you make something different if your child does not like what is being served?	4.24 (1.80)	2.79 (1.93)	**<0.001**	**0.78**
SFQ 59: Do you feed your child yourself if he/she does not eat enough?	0.85 (1.54)	0.73 (1.50)	0.569	0.08
SFQ 60: Do you give your child bottles of juice or milk in the car?	0.75 (1.64)	0.55 (1.30)	0.743	0.14
SFQ 61: Do you give your child bottles of juice or milk in his/her bed?	0.15 (0.77)	0.33 (1.03)	0.950	−0.20
SFQ 62: If your child wakes at night do you feed him/her to help him/her fall back to sleep?	0.17 (0.89)	0.22 (0.92)	0.691	−0.06

Note: SFQ—Stanford Feeding Questionnaire; PFP—Parental Feeding Problems; ARFID—Avoidant restrictive food intake disorder. Bold means statistical significant results.

**Table 3:** Comparison between children with ARFID and control children in noneating related psychometric scales.

Psychometric Tools	ARFID (*n* = 50) Mean (SD)	Control (*n* = 98) Mean (SD)	*p*-Value
SDQ-Total Difficulties Score	15.12 (7.09)	9.90 (6.42)	**<0.001**
SDQ-Externalizing Score	6.18 (3.90)	4.88 (3.826)	0.057
SDQ-Internalizing Score	8.94 (4.29)	5.01 (3.82)	**<0.001**
SRQ-Hedonic Score	2.05 (0.48)	2.21 (0.48)	**0.038**
SRQ-Aversive Score	1.89 (0.54)	1.73 (0.42)	0.113
Total Anxiety-Score (SCARED)	28.30 (19.71)	23.14 (16.14)	0.204

Note: ARFID-Avoidant restrictive food intake disorder; SDQ-Strengths and Difficulties Questionnaire; SRQ-Sensory Responsiveness Questionnaire; SCARED-Screen for Child Anxiety Related Disorders Questionnaire; SFQ-Stanford Feeding Questionnaire. Bold means statistical significant results.

**Table 4:** The potential of the parent reports to differentiate between children with ARFID and control children.

	Area under the Receiver Operating Characteristics Curve (ROC)	95% Confidence Interval	*p*-Value
SDQ-Total Difficulties score	0.716	0.628–0.805	**<0.001**
SDQ-Externalizing Difficulties score	0.596	0.500–0.692	0.058
SDQ-Internalizing Difficulties score	0.756	0.672–0.840	**<0.001**
SRQ-Hedonic score	0.598	0.694–0.501	0.054
SRQ-Aversive score	0.586	0.489–0.683	0.090
SCARED-Total Anxiety Score	0.569	0.465–0.673	0.172
SFQ-ARFID Score	0.939	0.895–0.983	**<0.001**
SFQ-Parental Feeding Problem (PFP) Score	0.624	0.531–0.717	**0.014**
Evaluated using the area under the Receiver Operating Characteristics Curve (ROC).			

Note: AEFID—Avoidant restrictive food intake disorder; SDQ—Strengths and Difficulties Questionnaire; SRQ—Sensory Responsiveness Questionnaire; SCARED—Screen for Child Anxiety Related Disorders Questionnaire; SFQ—Stanford Feeding Questionnaire. Bold means statistical significant results.

**Table 5:** Correlations between the different psychometric scales and the SFQ-ARFID Scale.

Psychometric Tools	SFQ-ARFID All Children (*n* = 148) r (*p*-Value)	SFQ-ARFID ARFID Children (*n* = 50) r (*p*-Value)	SFQ-ARFID Control Children (*n* = 98) r (*p*-Value)
SDQ-Total Difficulties Scale	**0.324 (<0.001)**	0.062 (0.673)	0.122 (0.232)
SDQ-Externalizing Scale	0.187 (0.24)	0.043 (0.770)	0.134 (0.190)
SDQ-Internalizing Scale	**0.353 (<0.001)**	0.101 (0.491)	0.086 (0.399)
SRQ-Hedonic Scale	−0.115 (0.164)	0.129 (0.373)	−0.034 (0.743)
SRQ-Aversive Scale	0.140 (0.090)	0.053 (0.717)	0.056 (0.584)
Total Anxiety-Score (SCARED)	0.119 (0.151)	−0.093 (0.522)	0.133 (0.192)
SFQ-PFP	**0.479 (<0.001)**	**0.339 (0.016)**	**0.513 (<0.001)**

Note. ARFID = avoidant/restrictive food intake disorder; SDQ = Strengths and Difficulties Questionnaire; SRQ = Sensory Responsiveness Questionnaire; SCARED = Screen for Child Anxiety Related Disorders Questionnaire; SFQ = Stanford Feeding Questionnaire; AUROC = area under receiver operating characteristics curve. Bold means statistical significant results.
